# Addition of 5-fluorouracil to doxorubicin-paclitaxel sequence increases caspase-dependent apoptosis in breast cancer cell lines

**DOI:** 10.1186/bcr1274

**Published:** 2005-06-22

**Authors:** Wainer Zoli, Paola Ulivi, Anna Tesei, Francesco Fabbri, Marco Rosetti, Roberta Maltoni, Donata Casadei Giunchi, Luca Ricotti, Giovanni Brigliadori, Ivan Vannini, Dino Amadori

**Affiliations:** 1 Division of Oncology and Diagnostics, Morgagni Pierantoni Hospital, Forlì, Italy; 2 Istituto Oncologico Romagnolo, Forlì, Italy

## Abstract

**Introduction:**

The aim of the study was to evaluate the activity of a combination of doxorubicin (Dox), paclitaxel (Pacl) and 5-fluorouracil (5-FU), to define the most effective schedule, and to investigate the mechanisms of action in human breast cancer cells.

**Methods:**

The study was performed on MCF-7 and BRC-230 cell lines. The cytotoxic activity was evaluated by sulphorhodamine B assay and the type of drug interaction was assessed by the median effect principle. Cell cycle perturbation and apoptosis were evaluated by flow cytometry, and apoptosis-related marker (p53, bcl-2, bax, p21), caspase and thymidylate synthase (TS) expression were assessed by western blot.

**Results:**

5-FU, used as a single agent, exerted a low cytotoxic activity in both cell lines. The Dox→Pacl sequence produced a synergistic cytocidal effect and enhanced the efficacy of subsequent exposure to 5-FU in both cell lines. Specifically, the Dox→Pacl sequence blocked cells in the G2-M phase, and the addition of 5-FU forced the cells to progress through the cell cycle or killed them. Furthermore, Dox→Pacl pretreatment produced a significant reduction in basal TS expression in both cell lines, probably favoring the increase in 5-FU activity. The sequence Dox→Pacl→48-h washout→5-FU produced a synergistic and highly schedule-dependent interaction (combination index < 1), resulting in an induction of apoptosis in both experimental models regardless of hormonal, p53, bcl-2 or bax status. Apoptosis in MCF-7 cells was induced through caspase-9 activation and anti-apoptosis-inducing factor hyperexpression. In the BRC-230 cell line, the apoptotic process was triggered only by a caspase-dependent mechanism. In particular, at the end of the three-drug treatment, caspase-8 activation triggered downstream executioner caspase-3 and, to a lesser degree, caspase-7.

**Conclusion:**

In our experimental models, characterized by different biomolecular profiles representing the different biology of human breast cancers, the schedule Dox→Pacl→48-h washout→5-FU was highly active and schedule-dependent and has recently been used to plan a phase I/II clinical protocol.

## Introduction

Breast cancer is still a leading cause of cancer death in women in the United States and Europe [1]. Adjuvant chemotherapy has been shown to provide disease-free and overall survival benefits for patients with node-positive breast cancer in large meta-analyses conducted by the Early Breast Cancer Trialists' Collaborative Group [2]. A study by Weiss *et al. *[3], however, showed that the overwhelming majority (80%) of node-positive patients relapse and die within 26 years of diagnosis despite the use of cyclophasphamide-methotrexate-5-fluorouracil (CMF)-based adjuvant chemotherapy. The development of drugs and strategies to improve relapse-free and overall survival therefore remains a high priority.

Anthracyclines are among the most active chemotherapeutic drugs for the treatment of breast cancer and anthracycline-containing regimens have an impact, albeit modest, on patient survival [2,4].

Paclitaxel (Pacl), belonging to the chemical class of taxanes, is capable of inducing *in vitro *apoptosis, independently of p53 status, through its microtubule-stabilizing activity and, as recently published, by inducing the release of cathepsin B from lysosomes [5]. From a clinical point of view, taxanes appear to be promising, although their real impact on the natural history of breast cancer has yet to be defined. A similar impact of Pacl on clinical response has been reported, however, for patients with wild-type or p53-mutated cancers [6-9], the latter representing 15% of *in situ *breast carcinomas and 50% of invasive disease [10]. Moreover, the loss of normal p53 function sensitizes *in vitro *cells to drug activity [11-13], hence the interest to use the taxane combination with other drugs as clinical therapy. The association of a taxane with an anthracycline is based on evidence that the two drugs have different mechanisms of action and toxicities and are not cross-resistant. Moreover, *in vitro *studies [14-17] on human cell lines and primary breast cancer cultures have shown a schedule-dependent interaction of the two drugs. Phase I and II studies with the doxorubicin (Dox)-Pacl sequence defined by preclinical studies have reported objective responses ranging from 40% to more than 90% [18,19], and a recent prospective phase III study on advanced breast cancer reported an impressive response rate (up to 94%) for the sequence, which was shown to prolong overall survival compared to the fluorouracil, epirubicin, cyclophosphamide (FEC) combination [20].

5-Fluorouracil (5-FU), one of the most important agents for the treatment of colorectal, head and neck, pancreatic and breast carcinomas, is a pro-drug that requires conversion to 5-fluoro-deoxyuridine-monophosphate (5FdUMP) and 5-fluoro-deoxyuridine-triphosphate (5FUTP) in cancer cells. Several *in vitro *studies on human solid tumor cell lines have demonstrated the positive and schedule-dependent interaction of Pacl and 5-FU [21-23]. A synergistic effect was obtained only when tumor cells were exposed to Pacl followed by antimetabolites. Conversely, simultaneous exposure to the two drugs or pretreatment with 5-FU reduced overall cell killing compared to Pacl alone. Specifically, it has been demonstrated that a short pretreatment with Dox increases the activity of Pacl and of the Pacl→gemcitabine sequence [24]. In the light of these preclinical and clinical observations, the present study aimed to investigate the cytotoxic effect produced by the combination of Dox, Pacl and 5-FU in human breast cancer cell lines and to define, at the preclinical level, the most effective treatment scheme.

## Materials and methods

### Cell lines

The study was performed on two human breast cancer cell lines: a commercial line (MCF-7; 40-h doubling time, obtained from the American Type Culture Collection (Rockville, MD), and a cell line established in our laboratory (BRC-230; 30-h doubling time) [25]. MCF-7 cells express estrogen receptors and bcl-2, harbor a wild-type *p53 *gene and lack pro-caspase-3. BRC-230 cells have no estrogen receptors, do not express bcl-2, contain a *p53 *gene mutation, and express pro-caspase-3. Culture medium was composed of DMEM/HAM F12 (1:1) supplemented with FCS (10% v/v), glutamine (2 mM) (Mascia Brunelli SpA, Milan, Italy) and insulin (10 μg/ml) (Sigma Aldrich, Milan, Italy). Cells were used in the exponential growth phase for all experiments.

### *In vitro *chemosensitivity assay

The sulforhodamine B (SRB) assay was used according to the method of Skehan *et al*. [26]. Briefly, 10^4 ^cells in 100 μl of medium/well were plated in 96-well flat-bottomed microtiter plates and 18 to 24 h after plating (an adequate time for exponential growth recovery) culture medium was replaced with fresh medium either containing or not containing the drugs. At the end of drug exposure, cells were fixed for 1 h and stained with 0.4% SRB (Sigma Aldrich) dissolved in 1% acetic acid for 30 minutes. The plates were then washed four times with 1% acetic acid to remove unbound stain, air-dried and solubilized in 100 μl of 10 mM unbuffered Tris base (tris(hydroxymethyl)aminomethane) solution. The optical density of treated cells was detected at 490 nm. Each sample was run in octuplet, and each experiment was repeated three times. Therefore, each experimental value in the graphs represents the median of 24 samples.

### Drugs and chemicals

Dox (Pharmacia Italia SpA, Milan, Italy) and 5-FU (Roche, Milan, Italy) were diluted in sterile saline solution, and Pacl (Bristol Meyers Squibb, Rome, Italy) was diluted in 95% ethanol. The drugs were then divided into aliquots and stored at -70°C. Drug stocks were freshly diluted in culture medium before each experiment. Z-LEHD-FMK (caspase-9 inhibitor) and Z-DEVD-FMK (caspase-3 inhibitor) (BD Biosciences Pharmingen, Milan, Italy) were solubilized in dimethylsulfoxide (DMSO) (Sigma Aldrich) and freshly diluted in culture medium at a concentration of 100 μM before each experiment. The final DMSO concentration never exceeded 1% and this condition was used as control in each experiment.

### Drug exposure

The drugs were tested singly at scalar concentrations of 0.001, 0.01, 0.1 and 1 μg/ml for Pacl and Dox, and 0.01, 0.1, 1 and 10 μg/ml for 5-FU. The exposure time to each single agent (4 h for Dox and 24 h for Pacl or 5-FU) was chosen from the dose inhibition rate curves and represented the time that produced the maximum effect. Control samples were processed as treated samples but in drug-free medium, and evaluation of the cytotoxic effect was performed immediately after the end of drug exposure. Each experiment testing the activity of the drugs singly and in combination was run in octuplet and repeated three times.

### Drug combinations

#### Dox/Pacl

Based on our previous results, in sequence experiments, cells were exposed to Dox for 4 h, after which the drug-containing medium was removed and cells were incubated for 24 h with fresh medium containing Pacl. In all the combination experiments, each drug was tested at the four different concentrations used for single drug exposure (0.001, 0.01, 0.1 and 1 μg/ml) at a 1:1 ratio.

#### Dox/Pacl/5-FU

Dox and Pacl were used as described above. After exposure, the medium was removed and cells were cultured in drug-free medium for 48 h, after which they were treated with 5-FU for 24 h. This scheme, derived from previous experimental studies, was considered as the most effective. In these combination experiments, the three drugs were tested at the four different concentrations used for single drug exposure (0.001, 0.01 and 0.1 and 1 μg/ml for Dox and Pacl and 0.01, 0.1, 1 and 10 μg/ml for 5-FU) at a 1:1:10 ratio.

The cytotoxic effect was evaluated immediately after the end of the three-drug exposure. Controls were processed as treated samples but in drug-free medium.

### Data analysis

The type of drug interaction was determined by the median effect principle according to the method of Chou and Talalay [27] and was applied for the three-drug treatment [28]. On the basis of this approach, the interaction between the three drugs was quantified by determining a combination index (CI) at increasing levels of cell kill. CI values lower than, equal to, or higher than 1 indicated synergy, additivity, or antagonism, respectively.

### Cell cycle perturbations

Cells (2 × 10^5^) were cultured in medium either containing or not containing (control) the cytotoxic drugs in sequence at a concentration of 0.1 μg/ml for Dox and Pacl and 1 μg/ml for 5-FU. After drug exposure, cells were harvested and stained in a solution containing RNAase (10 Ku/ml; Sigma-Aldrich), Nonidet P40 (0.01%; Sigma-Aldrich) and propidium iodide (1 μg/ml; Sigma-Aldrich). Samples were analyzed 30 to 60 minutes later by flow cytometry (Becton Dickinson Italia SpA, Milan, Italy). Data acquisition (10,000 events for each sample) was performed using CELLQuest software (Becton Dickinson Italia). Data were elaborated using Modfit (DNA Modelling System) software (Verity Software House Inc., Topsham, ME, USA) and expressed as fractions of cells in the different cell cycle phases. Samples were run in triplicate and each experiment was repeated three times.

### Apoptosis

Apoptosis was evaluated by flow cytometric analysis according to the previously described TUNEL assay procedure [29]. Briefly, the cells, treated as outlined above, were trypsinized, fixed, exposed to TUNEL reaction mixture and counterstained with propidium iodide before FACS analysis.

Trypsinized cells were treated with anti-Fas antibody (DakoCytomation Denmark A/S, Glostrup, Denmark) diluted 1:500 for 1 h at 4°C to evaluate Fas receptor expression by flow cytometry. Cells were then incubated with a goat-mouse FITC-conjugated secondary antibody (DakoCytomation). Flow cytometric data acquisition and analysis were performed using CELLQuest software (Becton Dickinson Italia). For each sample, 15,000 events were recorded.

### Western blot analysis

After the three-drug exposure, cells were treated according to the previously described western blot procedure [29]. The monoclonal antibodies used were anti-p53 (PAb 1801, Bioptica, Milan, Italy) 1:400, anti-bcl-2 (clone 124, Dako Corporation, Santa Barbara, CA, USA) 1:50, anti-p21 (clone DCS-60.2, Bioptica) 1:100, antithymidylate synthase (clone TS 106, Bioptica) 1:100 and anti-caspase-8 (clone 12F5, Alexis Biochemicals Corporation, Lausanne, Switzerland) 1:500. We used polyclonal antibodies for anti-caspases -3, -6, -7 and -9 (Cell Signaling Technology, Inc., Beverly, MA, USA), all diluted at 1:500, anti-apoptosis-inducing factor (AIF) (Chemicon International, Inc., Temecula, CA, USA) 1:500 and anti-bax (Pharmingen, San Diego, CA, USA) 1:1000. Actin polyclonal antibody (Sigma-Aldrich) was used as control to demonstrate equal loading. The samples were analyzed using QuantiScan software (Biosoft, Cambridge, UK).

## Results

### Cytotoxic activity

**Figure 1:**
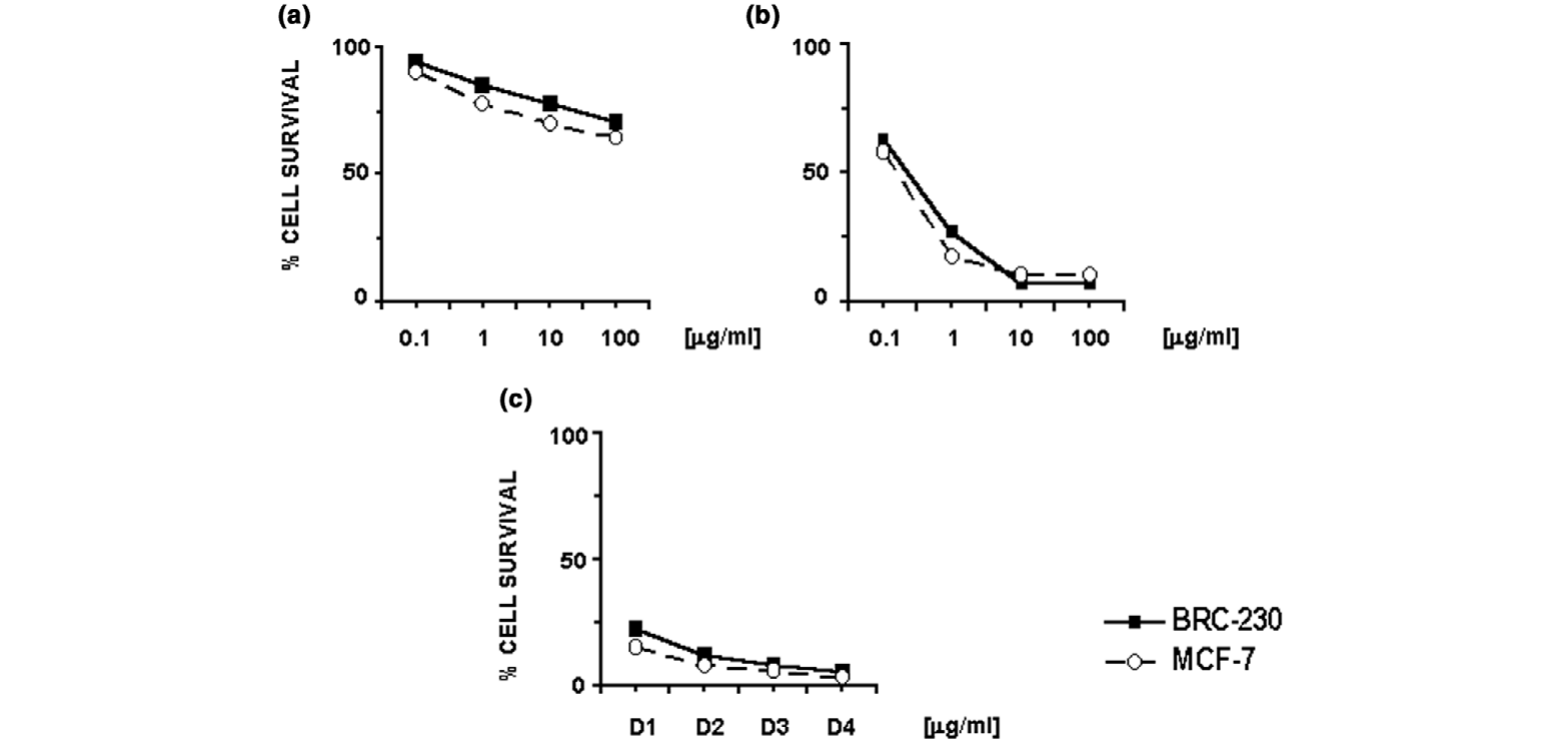
Effect of different drug treatments on the survival of MCF-7 and BRC-230 breast cancer cell lines. **(a) **5-FU, **(b) **Dox→Pacl sequence and **(c) **Dox→Pacl→5-FU treatment. D1, D2, D3 and D4 represent the doses of the three drugs used in the sequence (see Materials and methods). Each data point is the average of at least three independent experiments performed in octuplet. The standard deviation never exceeded 5%.

The cytotoxic activity of 5-FU, of the 4-h Dox→24-h Pacl sequence, and of the 4-h Dox→24-h Pacl→48-h wash-out→24-h 5-FU exposure is reported in Fig. 1. 5-FU showed a moderate cytotoxic activity in both cell lines. The Dox→Pacl sequence produced a strong cytocidal effect and an important synergism in MCF-7 and BRC-230 cell lines, as shown by Chou-Talalay analysis (Table 1). The synergistic effect dramatically increased, starting from the lowest doses, when cells were treated with 5-FU after the sequence anthracycline→taxane→48-h washout (Fig. 1).

### Cell cycle perturbations and apoptosis

**Figure 2:**
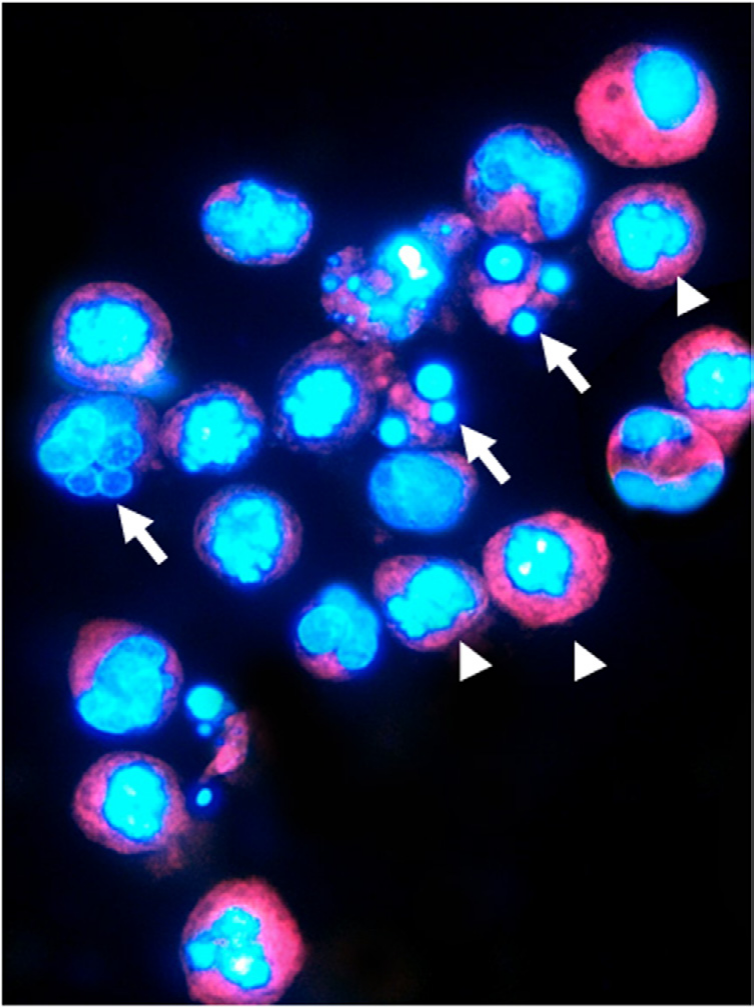
BRC-230 cells after Dox (0.1 μg/ml)→Pacl (0.1 μg/ml)→48-h washout→5-FU (1 μg/ml) treatment. Apoptotic nuclei stained with DAPI show intense fluorescence corresponding to chromatin condensation (arrow heads) and fragmentation (arrows).

A 4-h treatment with Dox or a 24-h exposure to 5-FU did not induce biologically relevant cell cycle perturbations (full recovery was observed after a 24-h culture in drug-free medium) or significant apoptosis (data not shown). Conversely, a 24-h treatment with Pacl produced a considerable increase of cells in G2-M phases, with a decrease in G1 phase, which further increased 24 h after drug removal and began to recover after 48 h. Moreover, about 10% and 15% of apoptotic cells were detected in BRC 230 and MCF-7 cell lines, respectively (data not shown). The Dox→Pacl sequence caused a dramatic block of cells in G2-M and a decrease in G1-S phases, which persisted up to 48 h after drug removal. In parallel, the fraction of apoptotic cells (Fig. 2) rose from 15% to 22% as washout time increased. This finding was similar in both cell lines (Table 2).

Cell cycle perturbations consisting of higher and lower fractions of cells in G2 and G1/S phases, respectively, persisted after exposure to the Dox→Pacl→5-FU sequence (Table 2), albeit to a lesser extent than that observed after the Dox→Pacl sequence. A statistically significant increase of up to 40% was observed in apoptotic cells.

**Figure 3:**
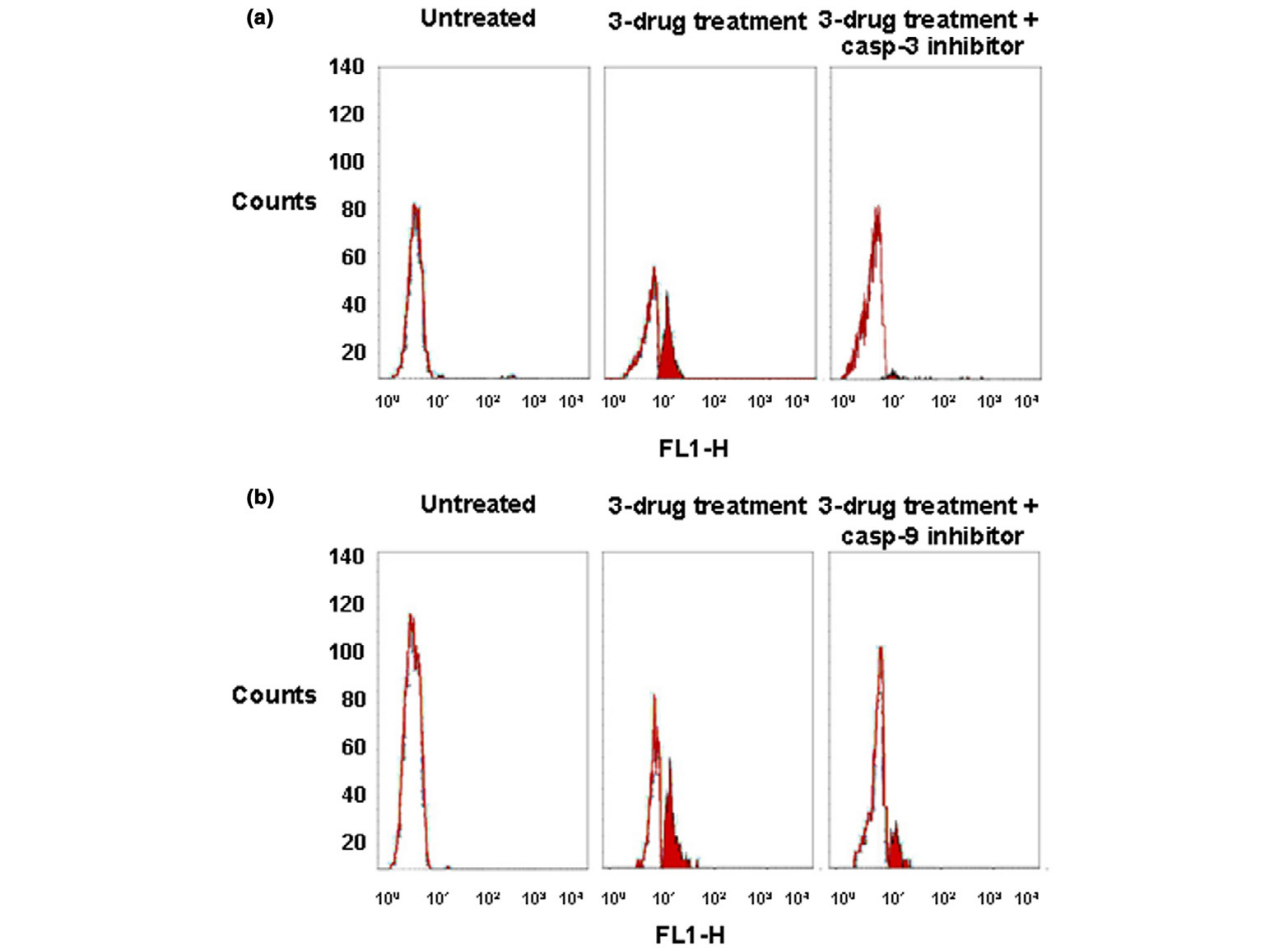
Inhibition of apoptosis. Results from TUNEL assay showing inhibition induced by Dox (0.1 μg/ml)→Pacl (0.1 μg/ml)→48-h washout→5-FU (1 μg/ml) treatment in **(a) **BRC-230 cells in the presence of caspase-3 (casp-3) inhibitor and **(b) **MCF-7cells in the presence of caspase-9 (casp-9) inhibitor.

The presence of caspase-9 inhibitor during exposure to the Dox→Pacl→5-FU sequence induced a reduction from 40% to 21% in the apoptotic cell fraction in the MCF-7 line (Fig. 3a). Similarly, the presence of caspase-3 inhibitor during the three-drug treatment caused a reduction in the apoptotic fraction from 37% to about 6% in BRC-230 cells (Fig. 3b).

### Apoptotic-related markers

**Figure 4:**
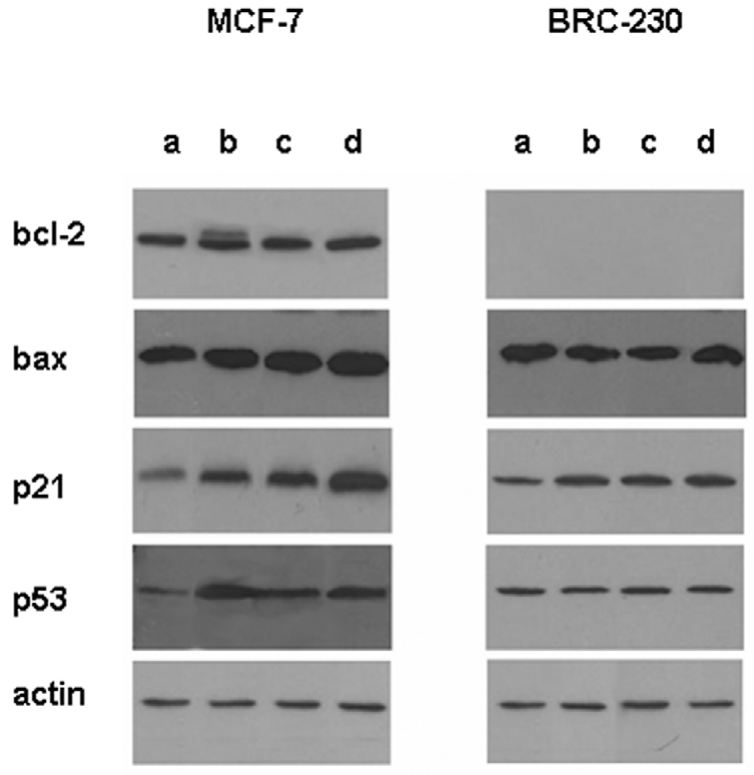
Protein levels of bcl-2, bax, p53 and p21 following different drug exposures. Protein (50 μg) was loaded for the controls and treated samples: **(a) **untreated cells; **(b) **Dox (0.1 μg/ml)→Pacl (0.1 μg/ml); **(c) **Dox (0.1 μg/ml)→Pacl (0.1 μg/ml)→48-h washout; **(d) **Dox (0.1 μg/ml)→Pacl (0.1 μg/ml)→48-h washout→5-FU (1 μg/ml).

In MCF-7 cells basally expressing bcl-2 and harboring wild-type p53, Dox or 5-FU did not have any effect on apoptotic-related markers, whereas Pacl caused bcl-2 phosphorylation as well as p53 overexpression (data not shown). Bcl-2 phosphorylation further increased after the Dox→Pacl sequence. An increase in pro-apoptotic bax, p21 and p53 expression was observed at the end of the two-drug sequence and was even more evident after the three-drug treatment (Fig. 4).

The BRC-230 cell line, lacking bcl-2 and with mutated p53, showed no induction of bax or p53 overexpression after any treatment. Conversely, Dox→Pacl treatment induced an increase in p21 expression that persisted 48 h after drug removal and was still evident at the end of the three-drug exposure (Fig. 4).

**Figure 5:**
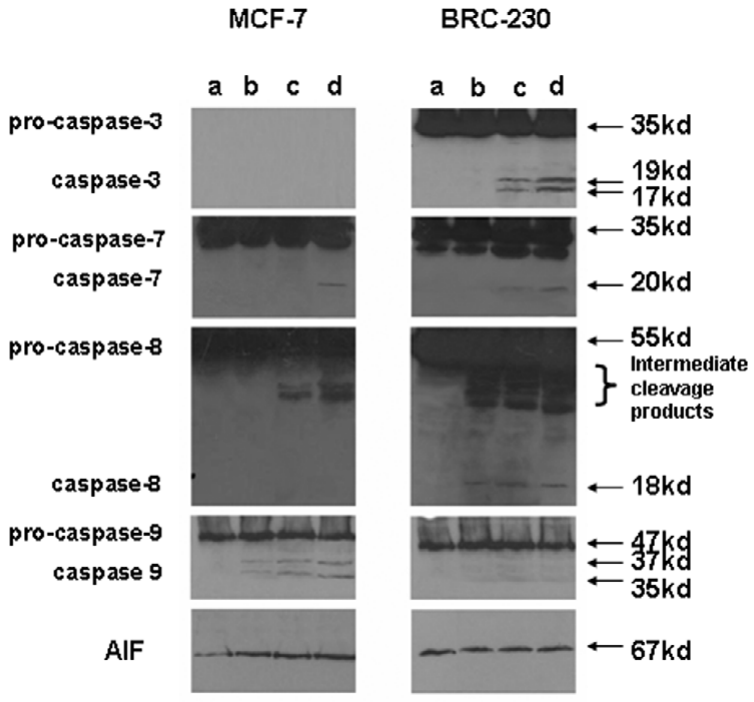
Western blot analysis of caspases and apoptosis-inducing factor (AIF) proteins following different treatments. Protein (50 μg) was loaded for the controls and treated samples: **(a) **untreated cells; **(b) **Dox (0.1 μg/ml)→Pacl (0.1 μg/ml); **(c) **Dox (0.1 μg/ml)→Pacl (0.1 μg/ml)→48-h washout; **(d) **Dox (0.1 μg/ml)→Pacl (0.1 μg/ml)→48-h washout→5-FU (1 μg/ml).

With regard to the caspase cascade, caspase-9 was activated in the MCF-7 cell line after the Dox→Pacl sequence and further increased at the end of the three-drug treatment. Similar behavior was observed for caspase-8, but its active 18 kDa fragment was not detected. Caspase-7 activation was induced after the three-drug sequence only (Fig. 5), whereas caspase-6 cleavage (data not shown) was not induced by any of the treatments. In parallel, a hyperexpression of AIF was observed at the end of both two- and three-drug treatments (Fig. 5).

**Figure 6:**
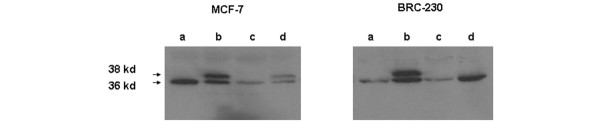
Western blot analysis of thymidylate synthase and ternary complex. **(a) **Untreated cells; **(b) **5-FU (1 μg/ml); **(c) **Dox (0.1 μg/ml)→Pacl (0.1 μg/ml); **(d) **Dox (0.1 μg/ml)→Pacl (0.1 μg/ml)→48-h washout→5-FU (1 μg/ml). 50 μg of protein were loaded for the controls and treated samples.

In BRC-230 cells, an induction of caspase-8, with its 18 kDa fragment, was observed at the end of the Dox→Pacl exposure and was still present after the three-drug treatment. Caspases -3 and -7 were activated 48 h after the end of Dox→Pacl treatment and persisted after the three-drug treatment. Conversely, none of the treatments induced caspase-9 (Fig. 5) or -6 expression (data not shown) or altered Fas expression (data not shown).

### Thymidylate synthase

Thymidylate synthase (TS) was basally expressed in both cell lines, but at higher levels in MCF-7 than in BRC-230, as seen by a clear western blotting band at 36 kDa (Fig. 6). 5-FU exposure induced a more evident increase in TS expression in the BRC-230 than in the MCF-7 cell line, as demonstrated by the appearance of an additional band of approximately 38 kDa.

Dox-Pacl treatment induced a significant decrease in TS expression in MCF-7 cells characterized by higher basal TS levels, and a slight reduction in low basal TS levels in the BRC-230 cell line. Exposure to 5-FU after Dox→Pacl treatment induced an increase in TS protein expression that was, however, lower than that induced by treatment with 5-FU alone, in both cell lines. Moreover, in BRC-230 cells, the three-drug treatment did not induce a detectable TS ternary complex (Fig. 6).

## Discussion

To date the clinical design of polychemotherapeutic protocols has mainly taken into account information derived from experimental studies on mechanisms of action of different agents and has favored combinations of drugs with complementary toxicities, but it has been clearly demonstrated that sequencing and timing of administration are important to optimize drug activity.

Our results confirmed the synergistic interaction of the Dox→Pacl treatment [14-16] and showed that this sequence enhances the efficacy of subsequent exposure to 5-FU, as observed in our previous studies using the antimetabolite gemcitabine [18,24]. In particular, we observed that cells, as expected, were trapped in the G2-M phase after the Dox→Pacl sequence, and the subsequent addition of 5-FU forced cells to progress through the cell cycle or killed them. In fact, the three-drug treatment doubled the percentage of apoptotic cells produced by the Dox→Pacl sequence in both experimental cell lines.

In MCF-7 cells, phosphorylation of the anti-apoptotic gene and persistent upregulation of pro-apoptotic markers was induced by the Dox→Pacl sequence and further enhanced by exposure to 5-FU. It can be hypothesized that the ensuing mitochondrial instability led to apoptosis through both the cytochrome-c-mediated activation of caspases -9 and -7 and the release of AIF. The important role played by AIF in the apoptosis process in MCF-7, which is lacking in caspase-3 [30], was demonstrated by the partial cell death inhibition observed in the presence of caspase-9 inhibitor during the three-drug treatment.

In the BRC-230 cell line, apoptosis was triggered by the activation of caspases -8 and -3 and, albeit to a lesser degree, caspase-7. In particular, the pivotal role of caspase-3 was highlighted by the almost complete suppression of apoptosis in the presence of caspase-3 inhibitor during the three-drug exposure.

TS protein was also modulated after the different treatments in both cell lines. TS, which converts dUMP to dTMP, is the primary target of fluoropyrimidine activity [31]. It represents the rate-limiting nucleotide in DNA synthesis and its overexpression has been shown to be associated with resistance to 5-FU-based treatments. Our results showed, as already reported in colon cancer cell lines [32], an increase in TS free protein and the formation of TS ternary complex following 5-FU exposure. This increase was more evident in the p53-mutated BRC-230 line than in wild-type p53 MCF-7 cells. Following the Dox→Pacl treatment, basal TS expression was significantly reduced in MCF-7 and also, albeit to a lesser degree, in BRC-230 cells. Subsequent exposure to 5-FU increased free TS expression without, however, reaching the levels observed after treatment with 5-FU alone. In particular, the increase involved only free TS in BRC-230 and was associated with the formation of the ternary complex, albeit fourfold lower than that induced by 5-FU alone, in MCF-7 cells.

In conclusion, apoptosis was observed after the Dox→Pacl sequence and was even more evident when followed by 5-FU, which induced an important apoptosis in cell lines characterized by different estrogen receptor status and apoptosis-related markers, both representative of the heterogeneous biology of clinical breast cancers. The efficacy of the antimetabolite was favored by an increase in TS levels following exposure to anthracycline and taxane.

## Conclusion

The present preclinical study permitted us to define the most effective three-drug sequence, providing the basis for the rationale of an ongoing phase I/II breast cancer clinical protocol in which the oral formulations of Dox and 5-FU are used to reduce toxicity and increase safety of treatment schemes.

## Abbreviations

5FdUMP = 5-fluoro-deoxyuridine-monophosphate; 5-FU = 5-fluorouracil; 5FUTP = 5-fluoro-deoxyuridine-triphosphate; AIF = apoptosis-inducing factor; CI = combination index; CMF = cyclophasphamide-methotrexate-5-fluorouracil; DMEM = Dulbecco's modified Eagle's medium; DMSO = dimethylsulfoxide; Dox = doxorubicin; FCS = fetal calf serum; FEC = fluorouracil, epirubicin, cyclophosphamide; Pacl = paclitaxel; SRB = sulforhodamine B; TS = thymidylate synthase.

## Competing interests

The authors declare that they have no competing interests.

## Authors' contributions

All the authors contributed equally to this study.

## Figures and Tables

**Table 1:** Combination index values induced by sequential treatments

		MCF-7			BRC-230		
		CI value at inhibition of			CI value at inhibition of		

Drug combination	Combination ratio	75%	90%	95%	75%	90%	95%
Dox→Pacl	1:1	0.3	0.3	0.4	0.1	0.2	0.2
Dox→Pacl→48-h washout→5-FU	1:1:10	0.0005	0.002	0.005	0.002	0.007	0.02

5-FU, 5-fluorouracil; CI, combination index; Dox, doxorubicin; Pacl, paclitaxel.

**Table 2:** Distribution of cells in the different cell cycle phases (%) and apoptotic cells (%) after different treatments

	MCF-7				BRC-230			
Treatment	G1	S	G2-M	Apoptosis	G1	S	G2-M	Apoptosis

Untreated cells	48	40	12	1	55	33	12	2
Dox→Pacl	6^a^	14^a^	80^a^	22^a^	10^a^	27^a^	63^a^	15^a^
Dox→Pacl→48-h washout	3^a^	16^a^	81^a^	17^a^	5^a^	11^a^	84^a^	15^a^
Dox→Pacl→48-h washout→5-FU	15^a^	49	36^a^	40^a^	20^a^	42^a^	38^a^	37^a^

^a^P < 0.05 by t-test. 5-FU, 5-fluorouracil; Dox, doxorubicin; Pacl, paclitaxel.
